# Regressions Fit for Purpose: Models of Locust Phase State Must Not Conflate Morphology With Behavior

**DOI:** 10.3389/fnbeh.2018.00137

**Published:** 2018-07-24

**Authors:** Swidbert R. Ott

**Affiliations:** Department of Neuroscience, Psychology and Behaviour, University of Leicester, Leicester, United Kingdom

**Keywords:** phenotypic plasticity, phenotypic integration, behavioral syndrome, phase change, logistic regression, multivariable analysis, desert locust, *Schistocerca gregaria*

## Abstract

Phenotypic plasticity often entails coordinated changes in multiple traits. The effects of two alternative environments on multiple phenotypic traits can be analyzed by multivariable binary logistic regression (LR). Locusts are grasshopper species (family Acrididae) with a capacity to transform between two distinct integrated phenotypes or “phases” in response to changes in population density: a solitarious phase, which occurs when densities are low, and a gregarious phase, which arises as a consequence of crowding and can form very large and economically damaging swarms. The two phases differ in behavior, physiology and morphology. A large body of work on the mechanistic basis of behavioral phase transitions has relied on LR models to estimate the probability of behavioral gregariousness from multiple behavioral variables. Mart́ın-Blázquez and Bakkali (2017; [10.1111/eea.12564]10.1111/eea.12564) have recently proposed standardized LR models for estimating an overall “gregariousness level” from a combination of behavioral and, unusually, morphometric variables. Here I develop a detailed argument to demonstrate that the premise of such an overall “gregariousness level” is fundamentally flawed, since locust phase transformations entail a decoupling of behavior and morphology. LR models that combine phenotypic traits with markedly different response times to environmental change are of very limited value for analyses of phase change in locusts, and of environmentally induced phenotypic transitions in general. I furthermore show why behavioral variables should not be adjusted by measures of body size that themselves differ between the two phases. I discuss the models fitted by Mart́ın-Blázquez and Bakkali (2017) to highlight potential pitfalls in statistical methodology that must be avoided when analysing associations between complex phenotypes and alternative environments. Finally, I reject the idea that “standardized models” provide a valid shortcut to estimating phase state across different developmental stages, strains or species. The points addressed here are pertinent to any research on transitions between complex phenotypes and behavioral syndromes.

## 1. Introduction

A central question in animal behavior is the extent to which individual differences in multiple behavioral traits are integrated together into behavioral syndromes (Sih et al., 2004; Dingemanse et al., 2010; Wolf and Weissing, 2012) or with other phenotypic dimensions such as morphology and physiology to form complex integrated phenotypes (Pigliucci, 2003; Murren, 2012; Armbruster et al., 2014; Kern et al., 2016). Phase change in locusts is a paradigmatic example of phenotypic plasticity and integration. Locusts can transform between two very distinct integrated phenotypes or phases in response to changes in population density (Pener and Simpson, 2009). This capacity for “phase change” underpins the formation and break-up of swarms (Simpson and Sword, 2008; Cullen et al., 2017). Locust populations that have experienced low density conditions for several generations comprise individuals in the solitarious phase, which is characterized by cryptic coloration and a cryptic behavioral strategy that includes sparse locomotor activity with a crepuscular diel pattern and an aversion to conspecifics. Conversely, populations that have experienced high density conditions for several generations comprise individuals in the gregarious phase, which shows distinct morphometric ratios, aposematic coloration, and a very different behavioral strategy that includes high levels of activity with a diurnal diel pattern and a propensity to be attracted toward conspecifics. The two phases also differ profoundly in metabolic and endocrine physiology and reproductive biology (Pener and Simpson, 2009).

Full phase transformation thus entails changes in many aspects of the phenotype that unfold over very different time scales: behavioral changes can occur within a few hours (Roessingh et al., 1993), whereas morphological changes can only occur over weeks or months, primarily as animals molt, with further changes accruing trans-generationally. Long-term phase state can be easily assessed by measuring morphological variables, and is therefore widely used in field surveys to inform locust control operations (Dirsh, 1951, 1953). The behavioral phenotype, however, cannot be inferred from morphometric measurements because it changes more quickly to reflect the recent history of population density. It also follows that mechanistic laboratory studies that target different aspects of phase change (behavioral, physiological, morphological) must each operate on an appropriate time scale. Most mechanistic studies to date have focussed on *behavioral phase change*, and have therefore operated on a time scale of hours or days (Anstey et al., 2009; Ma et al., 2011; Ott et al., 2012; Guo et al., 2015). In contrast, manipulations targeted at morphological phase traits would necessarily take weeks to manifest.

A trivially obvious prerequisite for any analysis of a specific phase trait is a meaningful measure of that trait. To this end, Mart́ın-Blázquez and Bakkali (2017) have recently proposed standardized logistic regression (LR) models for estimating “the gregariousness level” of individuals of the desert locust (*Schistocerca gregaria* Forskål) for adoption by the research community, together with suggestions for extending their approach to other locust species including the migratory locust (*Locusta migratoria* L.). I agree that standardized, accessible, open and transparent methods are needed (see also Cullen et al., 2017), but the models promoted by Mart́ın-Blázquez and Bakkali (2017) are, regrettably, fundamentally flawed. The present paper is not intended as a comprehensive and detailed critique of the paper by Mart́ın-Blázquez and Bakkali (2017); instead, I discuss specific conceptual and methodological shortcomings of that work that are germane to any research on transitions between complex phenotypes.

## 2. Methods

The raw data included in the Supporting Information of Mart́ın-Blázquez and Bakkali (2017) were analyzed in R (RRID:SCR_001905) version 3.3.3 (R Core Team, 2017) and RStudio (RRID:SCR_000432) version 1.0.143 (RStudio Inc., Boston, MA) running under OS X El Capitan version 10.11.6 (Apple Inc., Cupertino, CA). LR models were fitted using the function *glm* from the built-in R package *core* to exactly replicate the analysis in Mart́ın-Blázquez and Bakkali (2017). Additionally, LR models were fitted using the *lrm* function in package *rms*, version 5.1-1 (Harrell, 2017), to obtain bootstrap-corrected values for two measures of model performance: (1) Somers' *D*, a measure of the rank discrimination of the model; and (2) the intercept and slope of the calibration line, a measure of model calibration (Harrell, 2001). Coefficients of variation were compared using the test of Feltz and Miller (1996) as implemented in the function *asymptotic_test* from the package *cvequality*, version 0.1.1 (Marwick and Krishnamoorthy, 2017). All analyses are documented in the Supplementary Material of the present paper, which includes a report in PDF format and the .Rmd source code that generates the report reproducibly from the raw data of Mart́ın-Blázquez and Bakkali (2017).

**Figure 1:**
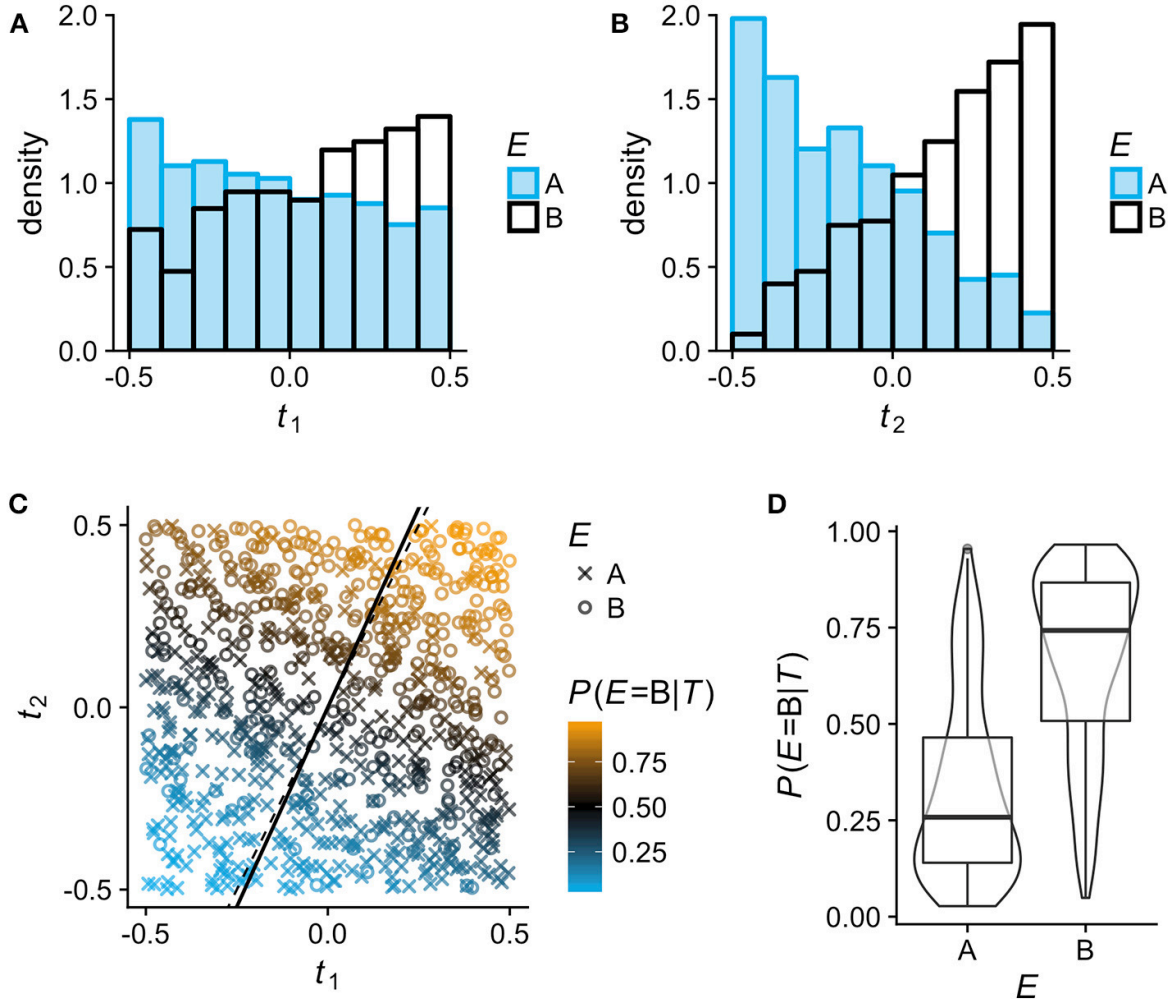
Binary logistic regression (LR) analysis of phenotypic differentiation between two environments A and B on simulated data (*N* = 800; see section Methods for details). In this example, the phenotypes comprise two uncorrelated continuous traits *T* = (*t*_1_, *t*_2_) and the association between the phenotypes *T* and the environment *E* follows logit(*P*(*E* = B|*T*)) = 2.5*t*_1_+5*t*_2_. To help visualize how LR estimates *P*(*E*|*T*) for any given *T* from the relative frequency of individuals from A and B, *t*_1_ and *t*_2_ are here simulated as uniformly distributed in [−0.5, 0.5], so that within this range, all phenotypes (trait combinations) are equally frequent in the total population. **(A,B)** Sample distributions of traits *t*_1_ and *t*_2_ in the two environments A and B: low values of *t*_1_ and *t*_2_ are more typical of individuals from environment A, high values more typical of individuals from B. **(C)** Visualization of the LR model fitted to the simulated data. Individuals from environment A and B are plotted as × and °, respectively; the color scale indicates their estimated *P*(*E* = B|*T*). LR entails projecting all phenotypes (trait combinations) on a single latent axis according to their estimated *P*(*E*|*T*). Any line orthogonal to the latent axis defines phenotypes that have the same *P*(*E*|*T*). The *solid line* is the latent axis as estimated by the fitted model (slope over *t*_1_ = 2.187); the true latent axis of the data generating model is shown by the *thin dashed line* (slope over *t*_1_ = 5/2.5 = 2). **(D)** The distributions of *P*(*E* = B|*T*) in the two environments A and B as estimated by the LR model.

For Figure 1, observations on *N* = 800 individuals with bivariate phenotypes *T* = (*t*_1_, *t*_2_) were simulated by independently sampling *t*_1_ and *t*_2_ from a continuous uniform distribution in [−0.5, 0.5]. The simulated observations were assigned to two environments (A, B) via a latent variable *y*′:

$$\begin{array}{lll} y^{\prime } & = & \beta_{0}+\beta_{1}t_{1}+\beta_{2}t_{2}+\epsilon \\ E & = & \text{B if }y^{\prime }>0,\\ E & = & \text{A otherwise}, \end{array}$$

with β_0_ = 0, β_1_ = 2.5, β_2_ = 5 and ϵ sampled from the logistic distribution with location μ = 0 and scale *s* = 1. The LR model *E*~*t*_1_+*t*_2_ was then fitted to the simulated data using the *lrm* function in *rms*, and the fitted model was used to predict *P*(*E* = *B*|*T*). The model fit gave the following estimates and standard errors (SE) for the model coefficients: ${\hat{\beta }}_{0}=-0.067,\text{ SE}=0.087;\;{\hat{\beta }}_{1}=2.26,\text{ SE}=0.319;\;{\hat{\beta }}_{2}=4.95,\text{ SE}=0.360$. The slope estimate of the latent axis over *t*_1_ (*solid line* in Figure 1C) was calculated as ${\hat{\beta }}_{2}/{\hat{\beta }}_{1}=2.187$. The code for generating Figure 1 is included in the Supplementary Material.

## 3. Phase transitions do not occur along a single latent axis

For the arguments that follow, it is useful to briefly explain how the effect of two alternative environments (A and B) on multi-trait phenotypes can be analyzed by multivariable binary logistic regression (LR; Figure 1). The LR approach entails modeling the association between phenotypes **T** and the environment *E* as *E*~**T**, with *E* as dependent variable, and estimating the probability that a given phenotype *T* is derived from environment B rather than A (or the other way around). In short, LR estimates *P*(*E*|*T*). Of all phenotypes with *P*(*E* = B|*T*) = 0.75, for example, 75% are expected to originate in environment B. A LR analysis thus projects all possible phenotypes (trait combinations) on a single latent axis (*solid line* in Figure 1C) according to their estimated *P*(*E*|*T*). An obvious but pertinent point is that very different phenotypes can be represented at the same position on this latent axis.

Mart́ın-Blázquez and Bakkali (2017) introduce the notion of an overall “gregariousness level” that encompasses all gregarious phase characteristics, be they molecular, behavioral, physiological, morphological or otherwise. I shall argue that this premise is conceptually misguided and of very limited value for any mechanistic analysis of phase change. In a series of influential papers, Simpson and colleagues introduced multivariable LR models to the analysis of behavioral phase change in the desert locust (Roessingh et al., 1993; Roessingh and Simpson, 1994; Simpson et al., 1999), and the approach has subsequently been extended to other locust species including *Locusta migratoria* (Guo et al., 2011; Ma et al., 2011) and *Chortoicetes terminifera* (Gray et al., 2009; Cullen et al., 2010, 2012). In all instances, the aim was to quantify *behavioral* gregariousness from multiple behavioral traits. Importantly, this approach assumes that phase-related behavioral traits change in concert during phase transformation, such that they can be interpreted as manifestations of a single latent *behavioral phase state*. In the desert locust, this assumption is not entirely uncontroversial (Tanaka and Nishide, 2013) but reasonably well-supported by experimental data (Rogers et al., 2014); in other species, the behavioral coherence during phase change would need to be explicitly tested.

The notion in Mart́ın-Blázquez and Bakkali (2017) of a “gregariousness level” that encompasses *all* gregarious phase traits is altogether different. They state that

“In order to successfully test functionality of a gene or molecule, quantitative measurements of the level of gregariousness are needed. Currently no valid molecular marker is available, thus the assessment of the degree of locust gregariousness is based on mathematical modeling.” (Mart́ın-Blázquez and Bakkali, 2017, abstract).

According to this notion, one is resorting to LR models only in lieu of a single reliable marker—which could, in principle, be any trait that differs between phases:

“There is a plethora of […] potential indicators of the state of a locust population. Apart from their developmental, survival, reproductive, immunological, and physiological differences, [the two] phases also differ in their morphology […] and behavior.” (Mart́ın-Blázquez and Bakkali, 2017, p. 21).

This presumes that all phase-related traits are tightly coupled at all times, so that any one can serve as a measure of the same latent “gregariousness.” It is a ground truth, however, that phase transformation entails a *decoupling* of different phase-related traits: some behaviors change within hours, whereas morphological changes take several generations to fully manifest (Pener and Simpson, 2009).

Mart́ın-Blázquez and Bakkali (2017) set out with a LR model that is based on a combination of morphometric and behavioral variables (model “*Sg_extended”*), and a version of this model (“*Sg_extended_corrected”*) is one of two put forward for adoption by the research community. To be clear, it is perfectly feasible to define a statistically sound LR model that incorporates morphometric, behavioral and other kinds of traits to predict the probability that a locust has a *long-term history* of high population density. For locusts that are undergoing phase transition, however, the “probability of gregariousness” (*P*_greg_) predicted by a morphological-behavioral “hybrid” model will be wholly uninformative. This becomes clear when one considers a long-term solitarious locust that has been crowded for one day. Its morphology will not have changed, but in important aspects of its behavior, such as locomotor activity, it will already be comparable with long-term gregarious locusts (Roessingh and Simpson, 1994). A first problem is that including both morphometric and behavioral predictors into a multivariable LR model does not guarantee that they will have equal weight—in fact, this outcome is unlikely. The estimation of the weights (coefficients) of the predictors is entirely data-dependent: each estimate reflects the association of the predictor with phase after adjusting for the associations of all other predictors with phase and, importantly, the degree of collinearity among predictors, with all estimates subject to stochasticity in the data sample to which the model is fitted. One or more predictor variables will dominate the prediction, and the dominant predictor(s) may be behavioral or morphological depending on the specific combination of predictors included in the model and the sample that the model is fitted to. For a one-day crowded locust, models that combine morphological and behavioral variables may therefore predict any *P*_greg_ value between near-zero (high probability of “solitariousness”) and near-one (high probability of “gregariousness”).

However, that entering predictors does not determine their weighting is almost a side point because, whatever the model prediction for our one-day crowded locust, it will in any case be inappropriate with respect to behavior, morphology, or both. If the model predicts a *P*_greg_ close to zero, it is obviously useless for detecting behavioral gregarisation. A value close to one would be entirely wrong as an estimate of morphometric phase state. Mart́ın-Blázquez and Bakkali (2017) may have hoped for a value of around 0.5—the prediction from a model in which behavioral and morphometric variables have equal weight—but this value too would be discordant with phase state: our one-day crowded locust is intermediate *neither* in morphology (it still has completely solitarious morphology) *nor* in behavior (it behaves gregariously). Overall phase state cannot be measured on a single latent axis but requires a multidimensional metric space—at least a plane spanned by behavior and morphology if we consider only those two aspects and were to assume that they can each be sensibly collapsed onto a single axis. Even this assumption is too simplistic, however, because different aspects of morphological phase change are known to be mechanistically decoupled from each other. For example, different components of the gregarious coloration can be induced by separate sensory stimuli (Lester et al., 2005).

## 4. Behavioral predictors should not be adjusted for body size

Mart́ın-Blázquez and Bakkali (2017) furthermore argue strongly that “speed-related behavioral variables” should be “normalized” for body size, criticize previous work for not having done so, and advocate dividing the “speed-related variables” by the length of the hind leg femur. Their argument is based on their reported correlations between “speed-related” and morphometric variables (Table S5 in the Supporting Information of Mart́ın-Blázquez and Bakkali, 2017). Here, it is necessary to distinguish between correlations *within* a phase and correlations *across* the two phases—a distinction that is not explicit in Mart́ın-Blázquez and Bakkali (2017). Gregarious desert locusts have shorter hind femora than solitarious locusts (Dirsh, 1951, 1953), yet they walk faster, more frequently and spend more time walking, and consequently they cover more ground during the assay duration (Ellis and Pearce, 1962; Roessingh et al., 1993; Rogers et al., 2014). Clearly, leg length does not explain these phase differences in locomotion, and the apparent correlations with hind femur length across the two phases are expected to be negative. The locusts used by Mart́ın-Blázquez and Bakkali (2017) are unusual in that they apparently have about 1.6 × *longer* hind legs in the gregarious phase (final instar nymphs; my analysis, Figure S1 and Tables S5, S6 in Supplementary Material). This may reflect a strain peculiarity that has not been reported in any other lab or wild strain, inappropriate husbandry, or a data labeling error; but whatever the cause it results in an unexpected *positive* across-phase correlation between *hind femur length* and *average speed* (my analysis; Spearman's ρ = 0.336; *N* = 66, *S* = 31832, *P* = 0.00589; Figure S2 in Supplementary Material).

For correlations *within* a phase, I have in most instances been unable to replicate the results in Mart́ın-Blázquez and Bakkali (2017) from the raw data provided in their Supporting Information (see Table S8 in Supplementary Material). For example, their Table S5 gives the correlation between *average speed* and *hind femur length* in gregarious nymphs as *r* = 0.442 (*N* = 51, *P* = 0.00128); I obtained *r* = −0.0612 (*N* = 51, *t*_49_ = −0.429, *P* = 0.670), which indicates that leg length has a negligible effect on locomotor speed. Also, several correlation coefficients in Mart́ın-Blázquez and Bakkali's (2017) Table 2 do not match those in their more detailed Table S5 (e.g., correlation between *hind femur length* and raw *erratic movement*, their Table 2: 0.215; their Table S5: −0.065; neither matches my calculation).

Nevertheless, one must concede that such correlations are plausible in principle. *If*, however, one were to commit to including both morphometric and behavioral predictors in a LR model, then adjusting upfront for a correlation between them is misguided because multivariable modeling already accounts for any correlations among predictors. While the individual associations of correlated predictors with the dependent variable cannot be resolved, such correlations do not affect predictive model performance (Harrell, 2001). Mart́ın-Blázquez and Bakkali's (2017) misconception about how predictors are weighted in multivariable model fits is apparent from their statement,

“To detect highly correlated variables that might reinforce or bias the model toward a particular trait, we carried out pairwise correlations […]” (Mart́ın-Blázquez and Bakkali, 2017, p.14):

Correlated predictors may lead to problems with model *estimation*, but they do not “reinforce” the model *predictions* because their joint information enters the prediction only once.

If, on the other hand, one rejects the inclusion of morphometric variables in the model on the grounds developed in the previous section, then dividing behavioral variables by femur length or any other measure of body size is misguided because it re-introduces morphometric characters into the model “through the back door.” Long-term solitarious locusts typically have longer legs. After 4–24 h of crowding, their locomotor characteristics will be virtually the same as those of long-term gregarious locusts but the legs will obviously be no shorter than before. If locomotor variables are divided by hind femur length, freshly gregarised locusts (with long legs) will yield values lower than those of gregarious locusts (with shorter legs) and will therefore be assigned an erroneously lower *P*_greg_ in what is intended as an assessment of pure behavioral gregariousness.

Adjusting behavior by morphology is ill-advised even if one considers only long-term solitarious and gregarious locusts. In Mart́ın-Blázquez and Bakkali's (2017) unusual data, where gregarious final instar nymphs have *longer* legs than their solitarious counterparts, a clear phase difference in *average speed* is obliterated after dividing by *hind femur length* (Figure S3 and Table S9 in Supplementary Material). This outcome is of course purely coincidental in the sense that the phase difference in *average speed* is not caused by the phase difference in leg length. But the consequence is that, in Mart́ın-Blázquez and Bakkali's (2017) data for final instar nymphs, raw *average speed* is a reasonably useful predictor of phase in a univariate LR model, whereas the corresponding model based on “normalized” *average speed* is hardly better than random guessing (Table S10 in Supplementary Material). Rather than improving predictive accuracy, as intended by Mart́ın-Blázquez and Bakkali (2017), the “normalization” annihilates the predictive power of *average speed* in their data.

To prove that dividing speed-related variables by hind femur length successfully “homogenizes” the variance, Mart́ın-Blázquez and Bakkali (2017) compared raw and “normalized” variances using Bartlett's tests, which are significant in all cases (Mart́ın-Blázquez and Bakkali, 2017, Table 3). These tests are meaningless, however, because variance depends on the measurement scale, and division by femur length changes the dimension and scale for only the “normalized” dataset. Bartlett's test is for comparing variances between different groups of data measured on the same scale, and will trivially give a significant result when applied to two versions of the same set of data measured on two different scales—dividing any set of values *X* by any factor *s*>1 will reduce the variance. The authors draw further erroneous conclusions from Bartlett's *K*^2^:

“It should be noted that if the animals are of similar size (the solitary samples used for this analysis), the normalization has a significant but clearly weaker effect (Table 3).” (Mart́ın-Blázquez and Bakkali, 2017, p.14)

Scale-dependency aside, however, *K*^2^ also depends on the sample size: the much lower values of *K*^2^ reported in their solitarious locusts reflect the almost 6-fold smaller sample (adults and final instar nymphs combined: *N* = 28 solitarious vs. *N* = 161 gregarious). An appropriate test would be comparing the sample estimates of the *coefficient of variation* $\hat{c_{\text{v}}}=s_{x}/\bar{x}$, a scale- and dimension-independent measure of relative dispersion (Feltz and Miller, 1996). In the example of the average speeds of solitarious and gregarious nymphs, division by femur length does not appreciably reduce the dispersion in Mart́ın-Blázquez and Bakkali's (2017) data (raw $\hat{c_{\text{v}}}=0.8227$, “normalized” $\hat{c_{\text{v}}}=0.7774$; *N* = 66 each for raw and “normalized,” Feltz-Miller statistic = 0.0915, *P* = 0.762). While some locomotion-related variables may conceivably correlate with body size, the evidence presented in Mart́ın-Blázquez and Bakkali (2017) is fallacious and the proposed remedy creates a problem rather than solving one.

## 5. Sample-size requirements for multivariable LR models

The most critical methodological failing of Mart́ın-Blázquez and Bakkali (2017), however, concerns the specific LR models that they put forward, which are based on a sample of 51 gregarious and 15 solitarious *S. gregaria* nymphs. How many predictors a LR model can reasonably accommodate is limited by the number of observations in the smaller group (here, 15 solitarious locusts; Harrell, 2001). The ratio of this “limiting sample size” to the number of regression coefficients (excluding the intercept) is known as “events per variable” (EPV; van Smeden et al., 2016). If EPV is low, the model will be unreliable; that is, it will not predict future observations as well as it appeared to predict on the present sample. Furthermore, there is an increased likelihood of “complete separation” of the two groups (here, of the two phases), in which case the model estimation fails altogether. The two models advocated in Mart́ın-Blázquez and Bakkali (2017), “*Sg_extended / Sg_extended_corrected”* and “*Sg_non-morphometric,”* have 13 and 10 predictors, respectively, which with 15 solitarious locusts means less than 2 EPV. Between 10 and 20 EPV are widely considered a minimum (Harrell, 2001), and while this is no hard and fast rule (van Smeden et al., 2016), fewer than 2 EPV is clearly too low for obtaining a reliable model fit.

This is demonstrated very instructively by inspection of the model fitting results in Mart́ın-Blázquez and Bakkali (2017). Replicating the LR fits in the same software as used in the paper (R, *glm* function) reproduces the numerical results with two-digit accuracy or better (Tables S13, S15, S20 in Supplementary Material). The slight discrepancies likely reflect platform differences in floating point arithmetic that only manifest when models are poorly estimable. Large apparent discrepancies are resolved as manuscript errors in Mart́ın-Blázquez and Bakkali (2017): First, in their Table 5, some variable names are switched: *average acceleration* should read *stop ratio; stop ratio* should read *turn ratio;* and *turn ratio* should read *average acceleration* (cf. their Table S5, where the labels are correct). Second, for models “*Sg_extended”* and “*Sg_non-morphometric,”* all coefficients and standard errors (SE) reported in the paper (Mart́ın-Blázquez and Bakkali, 2017, Table 5 and Table S5) are 10^3^ too high (Tables S13, S15 in Supplementary Material). This systematic error nevertheless accounts only in part for the unreasonably large coefficients and SEs reported for the two advocated models. Another contributing factor is the extreme scaling of the “normalized” speed-related variables (means between about 2 × 10^−4^ and 2 × 10^4^; Table S4 in Supplementary Material), which makes it hard to spot conspicuously large SEs that are indicative of collinearity problems.

After means-centring and scaling to sample standard deviation *s* = 1, re-fitting “*Sg_non-morphometric”* flags up pathologically large SE estimates for *average speed* (${\hat{\beta }}_{\text{as}}=-3.25$, SE = 75.9, *z* = −0.043, *P* = 0.966) and *average acceleration* (${\hat{\beta }}_{\text{aa}}=2.87$, SE = 75.8, *z* = 0.038, *P* = 0.970; Table S16 in Supplementary Material) that can be traced to near-perfect collinearity between them (*r* = 0.9998; Figure S4 in Supplementary Material). This must simply reflect a mistake in the measurement or calculation of one or both variables. More generally, however, the example highlights the importance of examining whether the estimated coefficients that one has obtained in the model fitting are *sensible*. At face value, ${\hat{\beta }}_{\text{as}}=-3.25$ would mean locusts that have a higher *average speed* are more likely to be *solitarious*, which would be very unexpected; but since in Mart́ın-Blázquez and Bakkali's (2017) data *average acceleration* is near-perfectly collinear with *average speed* and ${\hat{\beta }}_{\text{aa}}=2.87$, the two effectively cancel out. Furthermore, *final choice* (the side of the arena where the locust was at the end of the assay) encodes the sign of *last coordinate* in the arena, which makes *final choice* redundant. This resulted in a model fit where the coefficient is positive for *final choice*, but *negative* for *last coordinate* (although not significantly different from zero; Table S16 in Supplementary Material). It is thus important to examine whether the directions (signs) of the estimated coefficients are consistent both internally and with prior subject knowledge; where they are not, the model may be ill-specified.

After removing *average acceleration* and *final choice*, the fit and predictive performance of the “*Sg_non-morphometric”* model can be validated by bootstrapping, although model fitting still fails in about 15% of bootstrap samples due to divergence or singularity (*B* = 1000 bootstrap samples; Table S19 in Supplementary Material). The results indicate that the model's rank-discrimination is mediocre (bias-corrected Somers's *D* = 0.66, where 0 is no predictive power, 1 is perfect), but more importantly that the calibration is very poor: the bias-corrected calibration line has an intercept of 0.49 (where 0 is perfect, 1 is worst) and a slope of 0.45 (where 1 is perfect, 0 is worst), demonstrating the extreme overfitting that occurs with less than 2 EPV.

For the “*Sg_extended”*/“*Sg_extended_corrected”* model, *all* the coefficients are extremely large even after rescaling the predictors to *s* = 1, the SEs are astronomical and, consequently, all associated *P* values exceed 0.99 (Tables S13, S14 in Supplementary Material), as they do in Mart́ın-Blázquez and Bakkali (2017; Table S5 in their Supporting Information). These are diagnostic symptoms of a failed model fit. Replicating exactly the analysis in the paper shows that the *glm* function issues two warning messages when fitting “*Sg_extended”*: “*glm.fit: algorithm did not converge”* and “*fitted probabilities numerically 0 or 1 occurred,”* which together indicate that complete separation has occurred (see p. 11 in Supplementary Material). Mart́ın-Blázquez and Bakkali (2017) misinterpreted complete separation as excellent predictive accuracy—they describe their model as highly accurate because it “*detected all the 51 gregarious nymphs as gregarious with 100% probabilities and attributed 0% gregariousness probability to all our 15 solitary nymphs.”* The authors also considered a five-predictor model (“*Sg_low-redundancy”*) which, while still severely underpowered (3 EPV), did not result in complete separation. Of the three models in Mart́ın-Blázquez and Bakkali (2017), “*Sg_low-redundancy”* is the least deficient from a purely technical point of view. It has reasonable rank-discrimination (bootstrap bias-corrected Somers' *D* = 0.85) and shows considerable but tolerable overfitting (bootstrap bias-corrected linear calibration intercept = 0.09, slope = 0.80)—although the model fit fails in about 20% of bootstrap samples (Tables S20–S22). Because the authors did not consider calibration or rank-discrimination, they wrongly concluded that this model ‘*is not as accurate in predicting gregarious locusts as the “Sg_extended' model,”* and thus adopted “*Sg_extended.”* In attempting to validate the “*Sg_extended”* model on data from locusts reared at a range of intermediate population densities, Mart́ın-Blázquez and Bakkali (2017) found that it predicted extremely dichotomized probabilities that did not match the expected intermediate phase states. This inevitable consequence of the failed model fit cannot be “fixed” by shrinking the logit predictions *ad hoc* by an arbitrary “homogeneous correction” factor, which is what Mart́ın-Blázquez and Bakkali (2017) did to arrive at their final “*Sg_extended_corrected”* model that they recommended for uptake by the research community. Shrinkage methods that are grounded in statistical theory such as ridge and LASSO regression are available for handling the “many predictors/few samples” problem (Hastie et al., 2009). Alternatively, one may simply carry out a principal component analysis (PCA) on all candidate predictors, and then use the first few principal component scores as predictors in a LR model. This removes predictor redundancy and at the same time helps with reducing the number of predictors without *ad-hoc* or stepwise variable selection (Harrell, 2001). None of these methods, however, can overcome the limitation inherent to a sample size of 15, because nothing can generate information beyond what is provided by the sample.

## 6. Different stadia, strains and species need different models

Previous studies have used LR models that were based on samples from the same species, strain and developmental stage as those locust that the model was used to predict on. Studies on first-instar, final-instar or adult *S. gregaria* used models fitted to first- or final-instar nymphs or adults, respectively, of the same laboratory strain of that species (Roessingh et al., 1993; Islam et al., 1994; Boua¨ıchi et al., 1995); studies on different instars of *L. migratoria* used *L. migratoria* instar-specific models (Ma et al., 2011). The ultimate aim of Mart́ın-Blázquez and Bakkali (2017) are “standardized” models that can be applied across different laboratory strains, and ideally across different stadia and species (in their study, final instar nymphs and adults of *S. gregaria* and *L. migratoria*). Mart́ın-Blázquez and Bakkali (2017) gave explanations for why their efforts were only partially successful by their own lights. The validity of these explanations is limited by their small sample sizes and by fundamental statistical misconceptions that I have discussed above. Here I consider the broader question whether “standardized models” are a sound proposition in principle.

There are two distinct, although connected, aspects of “standardization” to Mart́ın-Blázquez and Bakkali's (2017) models. First, they are intended as community standards; second, they are based on behavioral predictors that have been “standardized” for morphometric differences. This second aspect was at least in part motivated by the hope that it would increase the validity of the models across different developmental stages, strains and species. Although Mart́ın-Blázquez and Bakkali (2017) concluded that their *S. gregaria* models did not perform well in *L. migratoria*, they recommended their “*Sg_non-morphometric”* model, which was fitted to final instars, for use in adults:

“For testing adults or the same nymphs at different time points (if they do not molt), we suggest using the “Sg_non-morphometric” model (that does not include morphometric variables).” (p. 23).

I refer to my earlier argument for why this model, although not containing overt morphometric variables, is nevertheless contaminated with morphometric information. Above, I have shown that “standardizing” behavior by morphology is inappropriate if the locusts that the model is fitted to are of the same developmental stage as those that the model is then used to predict on. “Standardizing” behavior by morphology is even more inappropriate if the model is then used to predict on locusts of a different developmental stage. This is readily seen when plotting Mart́ın-Blázquez and Bakkali's (2017) data for *average speed* over *hind femur length* for gregarious nymphs and adults: adults have distinctly longer hind femora (the distributions barely overlap), but their *average speeds* are *lower* in Mart́ın-Blázquez and Bakkali's (2017) data (Figure S5 and Table S23 in Supplementary Material). Consequently, dividing *average speed* by *femur length* increases, rather than reduces, the (now no longer purely behavioral) difference in “standardized average speed” between gregarious nymphs and adults (Figure S6 in Supplementary Material). Because behavioral differences between developmental stages, strains and species are not merely caused by morphology, they cannot be made disappear by dividing behavioral variables by femur length, or by any other morphometric.

Are standardized models without inappropriate “morphometric standardization” of behavioral predictors a valid proposition? Mart́ın-Blázquez and Bakkali (2017) give two motivations for their drive toward standardized models. First, it would do away with the need for each research group having to build their own model. This would save a great deal of effort, because building a model requires observations from a large number of “reference locusts” (certainly more than the 15 solitarious locusts used by Mart́ın-Blázquez and Bakkali, 2017). Second, the use of a common model would facilitate direct comparisons between results from different research groups.

At first, these arguments may appear attractive or even compelling. Their fallacy becomes apparent upon reflection on what we ask a standard model to do. We ask it to spare us the effort of collecting data for a different developmental stage, strain or species of locust; but these are the very data that are needed to ascertain the validity of the “standardized model” in that developmental stage, strain or species. One does not know a priori that a “standardized model” based on a sample of locusts from, e.g., strain A will adequately predict on locusts from a different strain B. Whether it does cannot be ascertained by validating the model in just a handful of locusts from B. To assess whether strain differences can be safely ignored, one needs an adequate number of observations on both phases from both strains under comparable laboratory conditions; fit a model that includes strain as an additional predictor variable together with all interactions between strain and the remaining predictors (Model 1); and fit the alternative model that does not include strain or any of its interactions (Model 2). In the symbolic form used to specify models in R, the models for *k* predictors *x*_1_, …*x*_*k*_ would be:

$$\begin{array}{ll} \text{Model }1:\;phase & \sim \left(x_{1}+x_{2}\ldots +x_{k}\right)*strain\\ \text{Model }2:\;phase & \sim x_{1}+x_{2}\ldots +x_{k} \end{array}$$

One can then compare the two models based on, e.g., AIC or a likelihood ratio test. Only if in an *adequately powered* analysis AIC were lower for Model 2 or if the test came out decisively “non-significant” (e.g., *P*>0.15) would we accept Model 2 as likely to predict well in both strains. Key here is *adequately powered:* to decide whether Model 2 is adequate, we need to have adequate data on both strains in the first place. This reveals the fallacy that a “standardized model” can save us the effort of collecting an adequately large sample in the strain that we want to predict on. The full model needs 2*k*+2 coefficients to be estimated (including the intercept), and by rule of thumb the minimum sample size would be about 15(2*k*+2) locusts of each phase, balanced with respect to strain. A strain-specific model requires *k*+1 coefficients to be estimated, and the minimum sample size would be about 15(*k*+1) locusts of each phase, of that strain. The conclusion from this is that validating an existing model in a new strain needs about as many locusts as building a new model.

Thus, a final and fundamental objection to the “standardized models” suggested by Mart́ın-Blázquez and Bakkali (2017) concerns the very proposition that one and the same model fit can be meaningfully generalized to different laboratory strains, let alone different developmental stages or species. This proposition fails to distinguish between a standardized *assay* and a standardized *fitted model*. There is a case for standardizing the assay conditions, although what aspects to standardize needs discussion—a behavioral arena for adults needs to be larger than one for first instar nymphs of the same species. The research community should agree on a standard set of predictor variables for LR models of behavioral phase state, and on transparent algorithms that define these variables. But the *fitted model* cannot be simply transplanted from one locust species, strain or developmental stage to another.

## 7. Conclusions

Although I appreciate Mart́ın-Blázquez and Bakkali's (2017) effort to promote open and transparent research, the advocated statistical procedures and models are conceptually and methodologically flawed and should not be adopted. The premise of an overall “level of gregariousness” that conflates morphological and behavioral phase characteristics is conceptually misguided, and statistical models based on this premise are of extremely limited use for the analysis of phase transitions. Standardizing behavior by body size creates a problem rather than solving one. Well-calibrated multivariable regression models require adequately large samples of locusts drawn from the population that one is working with, and the proposition that “standardized” models offer a shortcut to models that are valid across different developmental stages, strains or species needs to be rejected.

A broader lesson from this case study is that the investigator needs to clearly frame their question before embarking on the business of model fitting. In some sense, the kinds of model proposed by Mart́ın-Blázquez and Bakkali (2017) intend to cover all possible bases: with the ultimate goal maybe a model that works not only across multiple developmental stages, strains and species of locust, but also with all manner of traits—behavior, morphology, body color, gene expression and beyond; and thus necessarily across very different time scales, from hours to generations. In principle, one could of course use more complex and general models to describe plasticities in many different traits with very different response times. For example, one could conceive of models that predict whether a given adult locust resembles one crowded for an hour, a day, a week, a month or multiple generations; such models could, and indeed would need to, combine predictors across multiple “domains” (behavior, coloration, morphometrics, gene expression); but this would require huge *N* and the model would still only work for one developmental age at the time of observation (unless we get even more ambitious), and one direction of transformation, e.g., solitarious to gregarious. Before embarking on any such endeavor, however, one should ask: What is our question, and *what do we want the model to do for us?* If one is *a priori* interested either only in behavior or only in morphology, there is little point in specifying a model that includes “domain-extrinsic traits” that one is not interested in, or that one *knows* cannot change (such as morphology in adult locusts). Likewise, if one is interested in effects on specific time scales, it is unnecessary to specify a model that accommodates time scales from minutes to generations. Most modeling efforts are data-limited. Therefore, to ensure that the data gathering effort is spent on improving the accuracy of answers we want, we need to carefully match the scope of our models to our questions.

## Data availability statement

The datasets analyzed for this study can be found in the Supporting Information of Mart́ın-Blázquez and Bakkali (2017) doi:10.1111/eea.12564. The exact version of these data that was used as input for the analyses in the present study is included as Supplementary Material with this present paper.

## Author contributions

SO is the sole author of this article. SO conceived this article and the arguments that are put forward in it; carried out the statistical analyses, as documented in the Supplementary Material; and wrote the manuscript.

### Conflict of interest statement

The author declares that the research was conducted in the absence of any commercial or financial relationships that could be construed as a potential conflict of interest.

## Supplementary material

All analyses are documented in the Supplementary Material of the present paper, which includes a report in PDF format and the .Rmd source code that generates the report reproducibly from the raw data of Mart́ın-Blázquez and Bakkali (2017).
