# Supplementary material for: Regressions Fit for Purpose: Models of Locust Phase State Must Not Conflate Morphology With Behavior
Source: Front Behav Neurosci. 2018 Jul 24;12:137. doi: 10.3389/fnbeh.2018.00137 (PMC6066544; doi:10.3389/fnbeh.2018.00137)
Supplement: Supplementary file 1 [file Presentation_1.ZIP › Ott_Supplemental_Analyses.pdf]

Supplementary Material

Regressions Fit for Purpose: Models of Locust Phase State Must Not Conflate Morphology With Behaviour

Swidbert R. Ott

29 May 2018

Contents

The data **1**
Calculation of derived variables . . . 2
Defining subsets within the data . . . 3
Scaling of the predictors . . . 4

Relationships between body size, phase and behaviour **5**
Phase difference in hind femur length . . . 5
Sample size discrepancies . . . 6
Apparent errors in the calculations of correlation coefficients . . . 7
Correlation between femur length and average speed . . . 8
The effect of 'normalising' average speed on predictive power . . . 9
Coefficients of variation . . . 11

Logistic regression model fits **12**
The *'Sg_extended' / 'Sg_extended_corrected'* model . . . 12
The *'Sg_non-morphometric'* model . . . 13
The *'Sg_low-redundancy'* model . . . 16

Average speeds in gregarious nymphs and adults **17**

Code for Figure 1 **19**

References **20**

This documents the analyses that I have performed on the raw data from Martín-Blázquez and Bakkali (2017). The code for generating Figure 1 in the main manuscript is provided at the end. The `.Rmd` source file that generated this document is provided as part of the Supplementary Materials.

The data

The Supporting Information of Martín-Blázquez and Bakkali (2017) includes an Excel file (*eea12564-sup-0002-TableS5.xls*), which the authors refer to as "Table S5" in the paper. This Excel file comprises several worksheets. Their Table S5, worksheet *Sg_raw_data* contains the raw behavioural and morphometric data for *both* species of locusts, *Schistocerca gregaria* and *Locusta migratoria* (coded `Sg` and `Lm`, respectively, in the worksheet). The worksheet cannot be easily imported directly into R because it contains multiple header rows and additional interspersed rows with summary statistics. To import the data into R, I first saved the data in worksheet *Sg_raw_data* to a CSV file (*MartinBlazquez_Bakkali_raw_data.csv*).

Martín-Blázquez and Bakkali (2017) Table S5, worksheet *regression_morph_vs_bhvr* gives the correlation coefficients and associated P-values for the correlations between three morphometric variables, including *hind femur length,* and six 'speed-related' variables. Martín-Blázquez and Bakkali (2017) calculated the correlations both before and after dividing the 'speed-related' variables by *hind femur length.* I saved their values for the correlation coefficients between the raw 'speed-related' variables and *hind femur length* into a CSV file (*MartinBlazquez_Bakkali_femur_corrs.csv*) so that they can be compared with the values calculated from the raw data in worksheet *Sg_raw_data.*

```r
# Load data
All <- read.csv(file = "MartinBlazquez_Bakkali_raw_data.csv")
MBB.Cors <- read.csv(file = "MartinBlazquez_Bakkali_femur_corrs.csv")
```

Table S1 lists the variables that I have used in this analysis. I adopted the short variable names used in Martín-Blázquez and Bakkali (2017) in my R code, and I use their long names when referring to variables in the text. In this document, I use `typewriter` font for R variable names and *italics* for long variable names.

Table S1: Long and short names of variables used in this analysis. *) `VAR_f` indicates variable 'normalised' by division by *hind femur length.*; `VAR.s` and `VAR_f.s` are centred and scaled versions not used in Martín-Blázquez and Bakkali (2017).

Long name	R name	Variants*
hind femur length	`femur.l`	
pronotum dorsal length	`pronotum.l`	
head width	`head.w`	
pronotum-femur index	`PF`	
pronotum-head index	`PH`	
femur-head index	`FH`	
choice	`CH`	
elapsed time	`ET`	`ET_f`, `ET.s`, `ET_f.s`
total distance	`TD`	`TD_f`, `TD.s`, `TD_f.s`
average speed	`AS`	`AS_f`, `AS.s`, `AS_f.s`
average acceleration	`AA`	`AA_f`, `AA.s`, `AA_f.s`
stop ratio	`SR`	
last coordinate	`LC`	
choice/time	`CT`	`CT_f`, `CT.s`, `CT_f.s`
turn ratio	`TR`	
erratic movement	`EM`	`EM_f`, `EM.s`, `EM_f.s`

Calculation of derived variables

Following Martín-Blázquez and Bakkali (2017), I calculated three morphometric ratios:

- *pronotum-femur index,* `PF`: *pronotum dorsal length* divided by *hind femur length;*
- *pronotum-head index,* `PH`: *pronotum dorsal length* divided by *head width;*
- *femur-head index,* `FH`: *hind femur length* divided by *head width.*

Again following Martín-Blázquez and Bakkali (2017), I divided the 'speed-related' variables by *hind femur length* to 'normalise' the data. The 'speed-related' variables in Martín-Blázquez and Bakkali (2017) are *elapsed time* (`ET`), *total distance* (`TD`), *average speed* (`AS`), *average acceleration* (`AA`), *choice/time* (`CT`) and *erratic movement* (`EM`).

```r
# Morphometric ratios
All$PF <- All$pronotum.l/All$femur.l
All$PH <- All$pronotum.l/All$head.w
All$FH <- All$femur.l/All$head.w
```

```r
# 'Normalisation' by hind femur length
All$ET_f <- All$ET/All$femur.l
All$TD_f <- All$TD/All$femur.l
All$AS_f <- All$AS/All$femur.l
All$AA_f <- All$AA/All$femur.l
All$CT_f <- All$CT/All$femur.l
All$EM_f <- All$EM/All$femur.l
```

Defining subsets within the data

In Martín-Blázquez and Bakkali (2017), the highest crowding densities are 150 individuals per cage for final-instar nymphs and 300 per cage for adults. I selected the subset of data that are from *S. gregaria*, and within this subset, I selected

- final instar nymphs for which the rearing densities were "1" (solitarious) or "150" (gregarious), and
- adults for which the rearing densities were "1" (solitarious) or "300" (gregarious).

Table S2 shows the resulting sample sizes, which match those given in the *Material and method* section of Martín-Blázquez and Bakkali (2017), p.11.

```r
# Construct subsets of data
Sg <- subset(All, species=="Sg")
Sg.5th <- subset(Sg, stadium=="nymph" & density %in% c(1, 150))
Sg.Ad <- subset(Sg, stadium=="adult")  # no adults of intermediate density
```

```r
Sg.5th$phase <- ifelse(Sg.5th$density==150, "greg", "sol")
Sg.5th$phase <- factor(Sg.5th$phase, levels = c("sol", "greg"))

Sg.Ad$phase <- ifelse(Sg.Ad$density==300, "greg", "sol")
Sg.Ad$phase <- factor(Sg.Ad$phase, levels = c("sol", "greg"))

Sg.all <- rbind(Sg.5th, Sg.Ad)

table.2 <- with(Sg.all, table(phase, stadium))  # data for Table 2
```

Note that in the data included with Martín-Blázquez and Bakkali (2017), six *S. gregaria* adults have ***infinite*** *average speeds* (AS) and *average accelerations* (AA; Table S3). I set them to NA so they can be ignored later in the correlation tests.

```r
table.3 <- subset(Sg.all, AS == Inf)[, c(1:4, 7:8)]  # data for Table 3
Sg.all$AS[Sg.all$AS == Inf] <- NA
Sg.all$AA[Sg.all$AA == Inf] <- NA
```

Table S2: Sample sizes for solitarious and gregarious adults and final-instar nymphs in the supplemental data included in Martín-Blázquez and Bakkali (2017).

	adult	nymph
solitarious	13	15
gregarious	30	51

Table S3: Observations where *average speed* (`AS`) and *average acceleration* (`AA`) are infinite in the supplemental data included in Martín-Blázquez and Bakkali (2017).

	id	density	stadium	sex	AS	AA
155	MVI_1988	1	adult	male	Inf	Inf
161	MVI_0466	300	adult	female	Inf	Inf
165	MVI_0471	300	adult	male	Inf	Inf
170	MVI_0476	300	adult	female	Inf	Inf
182	MVI_0488	300	adult	male	Inf	Inf

Scaling of the predictors

After dividing the 'speed-related' variables by *hind femur length* (to "normalise" them as advocated in Martín-Blázquez and Bakkali 2017), their mean values are either very large or very small (Table S4). The extreme scaling makes it very hard to interpret the coefficients in the logistic regression models later on.

I therefore calculated a centred and scaled version of all continuous predictors while keeping the original values to replicate the model building in the paper.

```
# Calculate data for Table S4: predictor mean values
X <- with(Sg.5th,
          data.frame(PF, PH, FH, CH, ET_f, TD_f,
                     AS_f, AA_f, LC, CT_f, SR, TR, EM_f)
          )
table.4 <- as.data.frame(apply(X, 2, mean))  # data for Table S4
names(table.4) <- "mean"
```

```
# Create scaled versions of predictors
Sg.5th$PF.s <- scale(Sg.5th$PF)
Sg.5th$PH.s <- scale(Sg.5th$PH)
Sg.5th$FH.s <- scale(Sg.5th$FH)
# Sg.5th$CH.s <- scale(Sg.5th$CH) -- binary: do not scale
Sg.5th$ET.s <- scale(Sg.5th$ET) ;  Sg.5th$ET_f.s <- scale(Sg.5th$ET_f)
Sg.5th$TD.s <- scale(Sg.5th$TD) ;  Sg.5th$TD_f.s <- scale(Sg.5th$TD_f)
Sg.5th$AS.s <- scale(Sg.5th$AS) ;  Sg.5th$AS_f.s <- scale(Sg.5th$AS_f)
Sg.5th$AA.s <- scale(Sg.5th$AA) ;  Sg.5th$AA_f.s <- scale(Sg.5th$AA_f)
Sg.5th$LC.s <- scale(Sg.5th$LC)
Sg.5th$CT.s <- scale(Sg.5th$CT) ;  Sg.5th$CT_f.s <- scale(Sg.5th$CT_f)
Sg.5th$SR.s <- scale(Sg.5th$SR)
Sg.5th$TR.s <- scale(Sg.5th$TR)
Sg.5th$EM.s <- scale(Sg.5th$EM) ;  Sg.5th$EM_f.s <- scale(Sg.5th$EM_f)
```

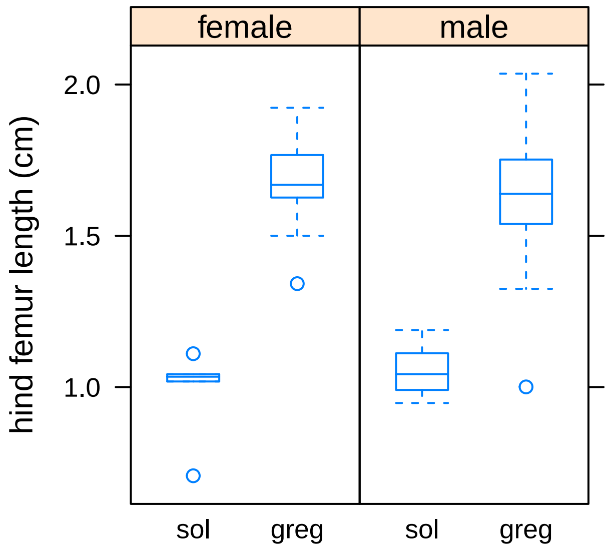

Figure S1: Difference in *hind femur length* between solitarious and gregarious final-instar nymphs. Data from Martín-Blázquez and Bakkali (2017).

Table S4: Mean values of the predictor variables in the logistic regression models of Martín-Blázquez and Bakkali (2017).

	mean
PF	0.4922
PH	1.4720
FH	3.0322
CH	0.4545
ET_f	27671.8415
TD_f	320.9800
AS_f	0.0140
AA_f	0.0002
LC	-8.2576
CT_f	0.0059
SR	0.4461
TR	0.3917
EM_f	10676.0299

Relationships between body size, phase and behaviour

This section relates to section *'Behavioural predictors should not be adjusted for body size'* in the main paper.

Phase difference in hind femur length

The *direction* of the phase difference in *hind femur length* in the Martín-Blázquez and Bakkali (2017) dataset is unexpected (Fig. 1): final-instar solitarious-phase nymphs have *shorter* hind femora than their gregarious counterparts (quartiles, Table S5; Wilcoxon two-sample rank sum test, Table S6).

```
sol <- with(subset(Sg.5th, phase=="sol"), quantile(femur.l))
greg <- with(subset(Sg.5th, phase=="greg"), quantile(femur.l))
table.5 <- rbind(sol, greg) ;  rownames(table.5) <- c("solitarious", "gregarious")
table.6 <- with(Sg.5th, wilcox.test(femur.l ~ phase))
```

Table S5: Quartiles of *hind femur length* in solitarious and gregarious final-instar nymphs. Data from Martín-Blázquez and Bakkali (2017).

	0%	25%	50%	75%	100%
solitarious	0.707	0.991	1.034	1.071	1.189
gregarious	1.001	1.552	1.654	1.753	2.036

Table S6: Wilcoxon two-sample rank sum test results: *hind femur length* in solitarious and gregarious final-instar nymphs. Data from Martín-Blázquez and Bakkali (2017).

Test statistic	P value	Alternative hypothesis
11	1.373e-08 * * *	two.sided

Sample size discrepancies

There are a number of internal discrepancies in Martín-Blázquez and Bakkali (2017) regarding the sample sizes used to calculate the correlations between morphometric and 'speed-related' variables.

- Martín-Blázquez and Bakkali (2017), legend for Table 2 states $N = 106$ and refers to Table S5 for details. Their table S5, worksheet *regression_morph_vs_bhvr,* states total $N = 109$ in the column labelled "All" for the "Pearson coefficients" / "Both" for the corresponding "P-values". In the raw data (worksheet *Sg_raw_data*), $N = 109$.
- In Martín-Blázquez and Bakkali (2017) Table S5, worksheet *regression_morph_vs_bhvr,* the N given for solitarious adults and nymphs do not match the N provided in the raw data, worksheet *Sg_raw_data* (Table S7).

More disconcerting are apparent errors in the calculations for the correlations (see next section).

Table S7: Mismatches (in bold) in sample sizes stated in Martín-Blázquez and Bakkali (2017) Table S5. [a]) as stated in worksheet *'regression morph vs bhvr'*; [b]) sample sizes in raw data (worksheet *'Sg raw data'*).

Group	*regression*[a]	*raw data*[b]
Gregarious Nymphs	51	51
Gregarious Adults	30	30
Solitarious Nymphs	**18**	**15**
Solitarious Adult	**10**	**13**
All gregarious	81	81
All solitarious	28	28
Total	109	109

Apparent errors in the calculations of correlation coefficients

The code chunk below calculates Pearson correlation coefficients between *hind femur length* and the six 'speed-related' variables from the raw data in Martín-Blázquez and Bakkali (2017), and compares them against the values in Martín-Blázquez and Bakkali (2017) Table S5, worksheet *regression_morph_vs_bhvr*. The majority of the values that I calculated do not match theirs (Table S8).

```
# calculate Pearson correlation coefficients between hind femur length
# and 'speed-related' variables from the raw data in @MBB.2017.
table.8 <- matrix(rep(NA, 6*7), nrow = 6)
row.names(table.8) <- c("ET", "TD", "AS", "CT", "AA", "EM")
colnames(table.8) <- c("greg.nymph", "greg.adult", "sol.nymph", "sol.adult",
                      "greg", "sol", "all")
for (phas in c("greg", "sol")) {
  for (stad in c("nymph", "adult")) {
    temp <- subset(Sg.all, phase==phas & stadium==stad)
    for (x in row.names(table.8)) {
      cor.test(~ temp[, x] + temp$femur.l, na.action="na.omit")$est ->
          table.8[x, paste(phas, stad, sep = ".")]
    }
  }
  temp <- subset(Sg.all, phase==phas)
  for (x in row.names(table.8)) {
    table.8[x, phas] <- cor.test(~ temp[, x] + temp$femur.l)$est
  }
}
for (x in row.names(table.8))
  table.8[x, "all"] <- cor.test(~ Sg.all[, x] + Sg.all$femur.l)$est

# In my dataframe `MBB.Cors`, the variable names are in a column.
# We need to remove this column so that the dataframe matches `table.8`:
row.names(MBB.Cors) <- MBB.Cors$var    # save var names into row names
MBB.Cors <- within(MBB.Cors, rm(var))  # and delete the var names column

# compare correlation coeffs with those in @MBB.2017 after rounding to 2 decimals:
no.match <- round(table.8, 2) != round(MBB.Cors, 2)
```

Table S8: Pearson correlation coefficients between *hind femur length* and 'speed-related' variables. Top half of table, as calculated from the raw data in Martín-Blázquez and Bakkali (2017). Values in red do not agree with those stated in Martín-Blázquez and Bakkali's (2017) Table S5, worksheet *regression_morph_vs_bhvr* ('MBB', bottom half of the table).

	greg.nymph	greg.adult	sol.nymph	sol.adult	greg	sol	all
ET	0.08	-0.09	-0.14	0.32	0.44	0.39	0.28
TD	0.24	-0.22	-0.23	0.22	0.08	0.27	0.09
AS	-0.06	0.05	0.14	-0.14	-0.29	0.19	-0.02
CT	-0.23	0.20	0.40	-0.18	-0.06	0.11	0.03
AA	-0.07	0.03	0.14	-0.15	-0.29	0.18	-0.04
EM	0.26	-0.27	-0.26	0.21	0.08	0.27	0.11
MBB: ET	-0.31	-0.09	0.74	0.35	0.36	0.65	0.36
MBB: TD	0.02	-0.22	0.06	0.17	0.07	0.30	0.09
MBB: AS	0.44	-0.22	-0.37	-0.11	0.06	0.20	0.08
MBB: CT	0.12	0.20	-0.22	-0.43	0.03	-0.37	0.03
MBB: AA	0.44	0.12	-0.37	-0.22	-0.12	-0.18	-0.09
MBB: EM	-0.08	0.17	0.34	-0.13	-0.04	-0.21	-0.06

Correlation between femur length and average speed

Figure S2 shows the relationship between *hind femur length* and *average speed* in final-instar nymphs of both phases. To test for a correlation *across* both phases, I used Spearman's rank correlations. To test for a correlation *within* the gregarious phase I used Pearson product moment correlations, to match the method of analysis in Martín-Blázquez and Bakkali (2017). The result within gregarious phase does not match that in Table S5 of Martín-Blázquez and Bakkali (2017) (cf. Table S8).

```
cor.test(~AS+femur.l, Sg.5th, method = "spear")
```

```
##
##  Spearman's rank correlation rho
##
## data:  AS and femur.l
## S = 31832, p-value = 0.005887
## alternative hypothesis: true rho is not equal to 0
## sample estimates:
##       rho
## 0.3355217
```

```
cor.test(~AS+femur.l, Sg.5th, subset = phase=="greg")
```

```
##
##  Pearson's product-moment correlation
##
## data:  AS and femur.l
## t = -0.42889, df = 49, p-value = 0.6699
## alternative hypothesis: true correlation is not equal to 0
## 95 percent confidence interval:
##  -0.3311576  0.2181045
## sample estimates:
##         cor
## -0.06115499
```

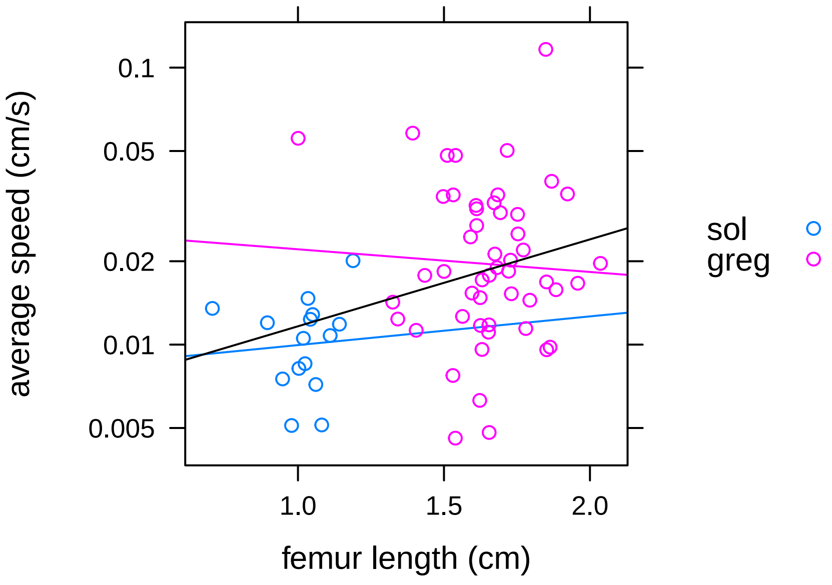

Figure S2: Relationship between *hind femur length* and *average speed*, with lines of best fit from OLS regressions. The black line shows the regression across the two phases, the blue and pink lines are from separate regression fits in solitarious and gregarious. Data from Martín-Blázquez and Bakkali (2017).

The effect of 'normalising' average speed on predictive power

Dividing by *hind femur length* (coincidentally) abolishes the phase difference in *average speed* in the data of Martín-Blázquez and Bakkali (2017) (Fig. S3; quartiles: Table S9). Consequently, raw *average speed* is a significant predictor of *phase* in a univariate LR model; 'normalisation' (division) by *hind femur length* abolishes predictive power (Table S10).

```
# temporary dataframe for plotting AS and AS_f in one figure
temp <- with(Sg.5th, data.frame(
  speed = c(AS, AS_f),
  kind = c(rep("raw", length(AS)), rep("norm", length(AS_f))),
  phase=rep(as.character(phase), 2))
  )
temp$phase <- factor(temp$phase, levels = c("sol", "greg"))
temp$kind <- factor(temp$kind, levels = c("raw", "norm"))

bwplot(speed ~ phase | kind, temp, pch="|",
       scales = list(y = list(log=T, equispaced.log=F)),
       ylab="average speed", xlab="phase")
```

```
# quartiles for Table S9
AS.sol <- with(subset(Sg.5th, phase=="sol"), quantile(AS))
AS.greg <- with(subset(Sg.5th, phase=="greg"), quantile(AS))
AS_f.sol <- with(subset(Sg.5th, phase=="sol"), quantile(AS_f))
AS_f.greg <- with(subset(Sg.5th, phase=="greg"), quantile(AS_f))

table.9 <- rbind(AS.sol, AS.greg, AS_f.sol, AS_f.greg)
```

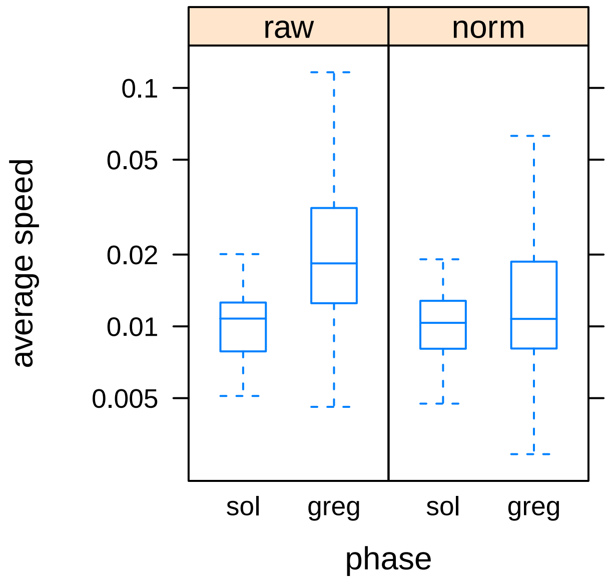

Figure S3: Phase difference in *average speed* of final instar nymphs before ('raw') and after ('norm') division by *hind femur length.* Data from Martín-Blázquez and Bakkali (2017).

Table S9: Quartiles of raw (`AS`) and 'normalised' (`AS_f`) *average speed* in solitarious and gregarious final-instar nymphs. Data from Martín-Blázquez and Bakkali (2017).

	0%	25%	50%	75%	100%
`AS`: solitarious	0.00510	0.00786	0.01078	0.01259	0.02009
`AS`: gregarious	0.00460	0.01250	0.01837	0.03137	0.11636
`AS_f`: solitarious	0.00474	0.00806	0.01035	0.01281	0.01911
`AS_f`: gregarious	0.00291	0.00807	0.01075	0.01869	0.06294

```
# logistic regression models fitted using `lrm` in `rms` package.
m1 <- lrm(phase ~ AS, Sg.5th)
LR.P <- 1 - pchisq(m1$stats["Model L.R."], df = 1)
m1.stats <- data.frame(Dxy = round(m1$stats["Dxy"], 4),
                       LR  = round(m1$stats["Model L.R."], 2), LR.P)

m2 <- lrm(phase ~ AS_f, Sg.5th)
LR.P <- 1 - pchisq(m2$stats["Model L.R."], df = 1)
m2.stats <- data.frame(Dxy = round(m2$stats["Dxy"], 4),
                       LR  = round(m2$stats["Model L.R."], 2), LR.P)

table.10 <- rbind(m1.stats, m2.stats)
```

Table S10: Comparison of univariate logistic regression models with raw (`AS`) or 'normalised' (`AS_f`) *average speed:* D_{xy}, Somers' D; LR χ^2, likelihood ratio χ^2 statistic.

	D_{xy}	LR χ^2	$P(>\chi^2)$
`phase ~ AS`	0.6301	17.08	0.0000358
`phase ~ AS_f`	0.1673	2.64	0.1044470

Coefficients of variation

The coefficient of variation (c_{v}) is defined as the ratio between the standard deviation σ and the mean μ; for a sample of x, it can be estimated from the sample standard deviation s_x and the sample mean $\bar{x}$:

$$\widehat{c_{\mathrm{v}}} = \frac{s_x}{\bar{x}}$$

To examine whether dividing ('normalising') *average speed* by *hind femur length* reduces the variation, as claimed by Martín-Blázquez and Bakkali (2017), I calculated $\widehat{c_{\mathrm{v}}}$ for *average speed* before (`AS`) and after (`AS_f`) division by *hind femur length* for nymphs of either phase (Table S11). I compared the two $\widehat{c_{\mathrm{v}}}$ values using the test of Feltz and Miller (1996) as implemented in the `cvequality` package (Table S12); see https://cran.r-project.org/web/packages/cvequality/vignettes/how_to_test_CVs.html

```
# Coefficients of variation: for Table S11
c.v_raw <- with(Sg.5th, sd(AS)/mean(AS))
c.v_nor <- with(Sg.5th, sd(AS_f)/mean(AS_f))

table.11 <- data.frame(Variable=c("AS", "AS_f"), c.v = round(c(c.v_raw, c.v_nor), 4))
```

```
# Feltz & Miller test: for Table S12
ad <- with(Sg.5th, asymptotic_test(
  c(AS, AS_f),
  c(rep("raw", length(AS)), rep("norm", length(AS_f))))
  )
table.12 <- as.data.frame(ad)
```

Table S11: Coefficients of variation for *average speed* before (`AS`) and after (`AS_f`) division by *hind femur length.*

Variable	$\widehat{c_{\mathrm{v}}}$
AS	0.8227
AS_f	0.7774

Table S12: Feltz and Miller (1996) test for equality of coefficients of variation before (`AS`) and after (`AS_f`) dividing *average speed* by *hind femur length.*

Test Stat.	P value
0.092	0.762

Logistic regression model fits

The *'Sg_extended'* / *'Sg_extended_corrected'* model

Attempting to fit the full 13-predictor *Sg_extended* model as given in Martín-Blázquez and Bakkali (2017) causes `glm.fit` to issue two serious warnings:

```
# predictors on original scale
m <- glm(phase ~ PF+PH+FH+CH+ET_f+TD_f+AS_f+AA_f+LC+CT_f+SR+TR+EM_f,
         family='binomial', data=Sg.5th)
```

```
## Warning: glm.fit: algorithm did not converge
```

```
## Warning: glm.fit: fitted probabilities numerically 0 or 1 occurred
```

```
table.13 <- signif(summary(m)$coefficients, 3)
```

The coefficients, standard errors, z values and P values from this failed model fit (Table S13) replicate the results in Martín-Blázquez and Bakkali (2017) with an accuracy of two digits or better, depending on the parameter in question. The discrepancies are likely due to hardware differences in floating point arithmetic that surface only when a model is ill-specified. To facilitate the interpretation of the coefficients and standard errors, I refitted the model to centred and scaled (sample standard deviation $s = 1$) predictors. The coefficients and standard errors are still astronomical (Table S14).

```
# predictors centred and scaled
m <- glm(phase ~ PF.s+PH.s+FH.s+CH+ET_f.s+TD_f.s+AS_f.s+AA_f.s+
           LC.s+CT_f.s+SR.s+TR.s+EM_f.s, family='binomial', data=Sg.5th)
```

```
## Warning: glm.fit: algorithm did not converge
```

```
## Warning: glm.fit: fitted probabilities numerically 0 or 1 occurred
```

```
table.14 <- round(summary(m)$coefficients, 3)
```

Table S13: Coefficients for *Sg_extended* model fitted to data from Martín-Blázquez and Bakkali (2017); predictor values on original scale.

	Estimate	Std. Error	z value	Pr(>|z|)
(Intercept)	-2.83e+04	3.81e+06	-0.00741	0.994
PF	5.10e+04	6.85e+06	0.00744	0.994
PH	-1.68e+04	2.26e+06	-0.00745	0.994
FH	9.36e+03	1.25e+06	0.00750	0.994
CH	1.03e+03	1.39e+05	0.00741	0.994
ET_f	-3.53e-03	8.26e-01	-0.00427	0.997
TD_f	-6.65e-01	1.66e+02	-0.00400	0.997
AS_f	7.89e+05	3.66e+08	0.00215	0.998
AA_f	-5.31e+07	2.44e+10	-0.00217	0.998
LC	-3.67e+00	5.12e+02	-0.00718	0.994
CT_f	1.89e+04	5.05e+06	0.00374	0.997
SR	-2.52e+02	1.36e+05	-0.00185	0.999
TR	-9.01e+02	1.59e+05	-0.00568	0.995
EM_f	2.91e-02	3.74e+00	0.00777	0.994

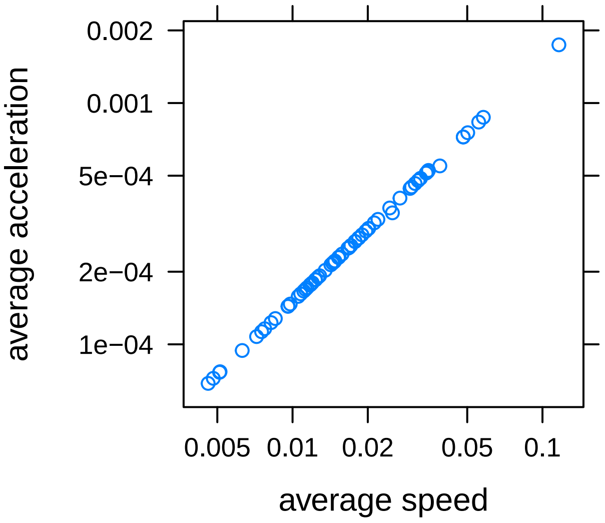

Figure S4: Near-perfect collinearity between *average speed* (`AS`) and *average acceleration* (`AA`) in the data from Martín-Blázquez and Bakkali (2017). Original values on log-scale axes.

Table S14: Coefficients for *Sg_extended* model fitted to data from Martín-Blázquez and Bakkali (2017); predictor values centred and scaled to $\bar{x} = 0, s_x = 1$.

	Estimate	Std. Error	z value	Pr(>|z|)
(Intercept)	18.484	30730.32	0.001	1.000
PF.s	3228.343	433873.93	0.007	0.994
PH.s	-1707.928	229296.97	-0.007	0.994
FH.s	3623.120	482840.14	0.008	0.994
CH	1026.467	138614.72	0.007	0.994
ET_f.s	-122.662	28748.73	-0.004	0.997
TD_f.s	-137.211	34276.21	-0.004	0.997
AS_f.s	8607.416	3995564.00	0.002	0.998
AA_f.s	-8691.402	3996676.28	-0.002	0.998
LC.s	-478.830	66680.08	-0.007	0.994
CT_f.s	174.111	46518.21	0.004	0.997
SR.s	-42.994	23256.38	-0.002	0.999
TR.s	-117.582	20707.40	-0.006	0.995
EM_f.s	175.235	22539.59	0.008	0.994

The *'Sg_non-morphometric'* model

Fitting the *'Sg_non-morphometric'* model replicates the results in Martín-Blázquez and Bakkali (2017) with an accuracy of two digits or better, depending on the parameter in question (Table S15). Again, I also fitted the model to centred and scaled ($s = 1$) predictors to facilitate the interpretation of the coefficients and standard errors (Table S16).

`AS_f` and `AA_f` have enormous standard errors (SE) even after scaling the predictors to $s = 1$. These are caused by a near-perfect collinearity between *average speed* (`AS`) and *average acceleration* (`AA`) (Fig. S4), which also manifests in their enormous VIFs (Table S17).

```r
# predictors on original scale
m <- glm(phase ~ CH+ET_f+TD_f+AS_f+AA_f+LC+CT_f+SR+TR+EM_f,
         family='binomial', data=Sg.5th)
table.15 <- signif(summary(m)$coefficients, 3)

# predictors centred and scaled
m <- glm(phase ~ CH+ET_f.s+TD_f.s+AS_f.s+AA_f.s+LC.s+CT_f.s+SR.s+TR.s+EM_f.s,
         family='binomial', data=Sg.5th)
table.16 <- round(summary(m)$coefficients, 3)
table.17 <- vif(m)  # variance inflation factors
```

After removing *average acceleration* (`AA_f.s`) from the model, the VIFs flag up a further collinearity issue between *last coordinate* (`LC`) and *choice* (`CH`). These two variables are redundant because `CH` is identical with the sign of `LC`. Once `CH` is also removed, the VIFs are still quite high (Table S18).

```r
m <- update(m, .~. - AA_f.s)  # remove `AA_f.s
# vif(m)  # variance inflation factors

m <- update(m, .~. - CH)  # also remove `CH`
table.18 <- vif(m)  # variance inflation factors
```

Table S15: Coefficients for *Sg_non-morphometric* model fitted to data from Martín-Blázquez and Bakkali (2017); predictor values on original scale.

	Estimate	Std. Error	z value	Pr(>|z|)
(Intercept)	1.12e+01	9.27e+00	1.2100	0.2280
CH	8.45e+00	3.83e+00	2.2100	0.0272
ET_f	5.30e-06	4.36e-05	0.1210	0.9040
TD_f	-2.13e-02	9.48e-03	-2.2500	0.0246
AS_f	-2.98e+02	6.95e+03	-0.0428	0.9660
AA_f	1.75e+04	4.63e+05	0.0378	0.9700
LC	-1.83e-02	1.60e-02	-1.1400	0.2530
CT_f	-6.45e+01	2.25e+02	-0.2870	0.7740
SR	-1.47e+01	1.11e+01	-1.3300	0.1840
TR	-7.52e+00	1.05e+01	-0.7190	0.4720
EM_f	4.35e-04	1.97e-04	2.2100	0.0274

Table S16: Coefficients for *Sg_non-morphometric* model fitted to data from Martín-Blázquez and Bakkali (2017); predictors centred and scaled to $\bar{x} = 0, s_x = 1$.

	Estimate	Std. Error	z value	Pr(>|z|)
(Intercept)	-1.108	1.401	-0.791	0.429
CH	8.449	3.826	2.208	0.027
ET_f.s	0.183	1.515	0.121	0.904
TD_f.s	-4.393	1.955	-2.247	0.025
AS_f.s	-3.250	75.870	-0.043	0.966
AA_f.s	2.868	75.770	0.038	0.970
LC.s	-2.389	2.089	-1.144	0.253
CT_f.s	-0.593	2.066	-0.287	0.774
SR.s	-2.514	1.892	-1.328	0.184
TR.s	-0.982	1.366	-0.719	0.472
EM_f.s	2.623	1.189	2.206	0.027

Table S17: Variance inflation factors (VIF) for the predictors in model *Sg_non-morphometric* fitted to scaled and centred data from Martín-Blázquez and Bakkali (2017).

CH	ET_f.s	TD_f.s	AS_f.s	AA_f.s	LC.s	CT_f.s	SR.s	TR.s	EM_f.s
13.84	4.295	13.36	21714	21757	19.26	6.761	9.325	7.128	6.579

Table S18: Variance inflation factors (VIF) for the predictors in model *Sg_non-morphometric* fitted to scaled and centred data from Martín-Blázquez and Bakkali (2017), after removing `AA_f.s` and `CH` from the model.

ET_f.s	TD_f.s	AS_f.s	LC.s	CT_f.s	SR.s	TR.s	EM_f.s
5.194	11.9	5.428	3.327	4.708	7.569	5.31	6.838

I refitted the model with the `lrm` function, package `rms`, to validate the model's indices of fit by bootstrap resampling ($B = 1000$; Table S19). Even with the two most redundant predictors removed, the model fit fails in 16% of the bootstrap samples. Note that there are still only $15/8 < 2$ 'events per variable'.

```
m <- lrm(phase ~ ET_f.s+TD_f.s+AS_f.s+LC.s+CT_f.s+SR.s+TR.s+EM_f.s,
         data=Sg.5th, x=T,y=T)
table.19 <- validate(m, B=1000)
```

```
##
## Divergence or singularity in 161 samples
```

Table S19. Model *'Sg_non_morphometric'* after removal of *average acceleration* and *final choice*; indices of model fit from bootstrap resampling ($B = 1000$). n is the number of successful bootstrap samples.

Index	Original Sample	Training Sample	Test Sample	Optimism	Corrected Index	n
D_{xy}	0.8248	0.8948	0.7331	0.1617	0.6631	839
R^2	0.5797	0.7008	0.4392	0.2615	0.3182	839
Intercept	0.0000	0.0000	0.4906	−0.4906	0.4906	839
Slope	1.0000	1.0000	0.4490	0.5510	0.4490	839
$E_{\max}$	0.0000	0.0000	0.2804	0.2804	0.2804	839
D	0.4649	0.6060	0.3289	0.2770	0.1879	839
U	−0.0303	−0.0303	0.6713	−0.7016	0.6713	839
Q	0.4952	0.6363	−0.3424	0.9787	−0.4835	839
B	0.0950	0.0672	0.1193	−0.0521	0.1471	839
g	3.1115	7.4759	2.3064	5.1695	−2.0580	839
g_p	0.2939	0.3150	0.2507	0.0643	0.2296	839

The '*Sg_low-redundancy*' model

Fitting the five-predictor *Sg_low-redundancy* model to the data in Martín-Blázquez and Bakkali (2017) replicates exactly the results in their Table S5, worksheet *model_coef_stats* (Table S20). The low VIFs (Table S21) demonstrate that this model does not suffer from the collinearity issues that afflict *Sg_extended* and *Sg_non-morphometric*, which explains the exact numeric replication (machine architecture related differences surface only when the model is ill-specified). However, the model fit still fails in about 20% of bootstrap samples (Table S22).

```
m <- glm(phase ~ PH+FH+CH+TD_f+SR, family='binomial', data=Sg.5th)
table.20 <- round(summary(m)$coefficients, 3)
table.21 <- vif(m)  # variance inflation factors

m <- lrm(phase ~ PH.s+FH.s+CH+TD_f.s+SR.s, data=Sg.5th, x=T,y=T)
table.22 <- validate(m, B=1000)
```

```
##
## Divergence or singularity in 202 samples
```

Table S20: Coefficients for *Sg_low-redundancy* model fitted to data from Martín-Blázquez and Bakkali (2017); predictor values on original scale.

	Estimate	Std. Error	z value	Pr(>|z|)
(Intercept)	-24.480	10.264	-2.385	0.017
PH	14.940	6.805	2.196	0.028
FH	2.765	1.770	1.562	0.118
CH	2.465	1.427	1.727	0.084
TD_f	-0.006	0.004	-1.740	0.082
SR	-5.307	3.572	-1.486	0.137

Table S21: Variance inflation factors (VIF) for the predictors in model *Sg_low-redundancy* fitted to the original values from Martín-Blázquez and Bakkali (2017).

PH	FH	CH	TD_f	SR
1.155	1.25	1.656	1.593	1.172

Table S22. Model '*Sg_low_redundancy*': indices of model fit from bootstrap resampling ($B = 1000$). n is the number of successful bootstrap samples.

Index	Original Sample	Training Sample	Test Sample	Optimism	Corrected Index	n
D_{xy}	0.8771	0.8978	0.8668	0.0310	0.8462	798
R^2	0.7320	0.7587	0.6757	0.0830	0.6489	798
Intercept	0.0000	0.0000	0.0887	−0.0887	0.0887	798
Slope	1.0000	1.0000	0.7967	0.2033	0.7967	798
$E_{\max}$	0.0000	0.0000	0.0661	0.0661	0.0661	798
D	0.6414	0.6810	0.5748	0.1063	0.5352	798
U	−0.0303	−0.0303	0.2330	−0.2633	0.2330	798
Q	0.6717	0.7113	0.3417	0.3696	0.3021	798
B	0.0517	0.0481	0.0670	−0.0189	0.0706	798
g	3.6539	6.6752	3.4031	3.2721	0.3817	798
g_p	0.3265	0.3260	0.3152	0.0108	0.3158	798

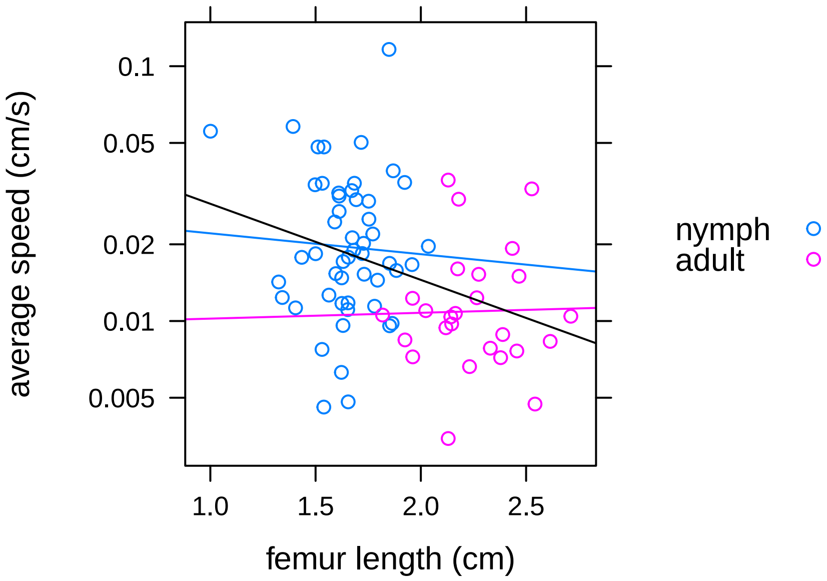

Figure S5: Relationship between *hind femur length* and *average speed* in gregarious nymphs and adults, with lines of best fit from OLS regressions. The black line shows the regression across the two stages, the blue and pink lines are from separate regression fits in adults and nymphs. Data from Martín-Blázquez and Bakkali (2017).

Average speeds in gregarious nymphs and adults

Gregarious adults have much longer hind femora than gregarious final-instar nymphs, but their *average speeds* in the data from Martín-Blázquez and Bakkali (2017) are lower (Fig. S5, Table S23). Therefore, dividing *average speed* by *hind femur length* ***increases*** the difference in *'average speed'* between gregarious final instar nymphs and adults (Fig. S6).

```
Sg.greg <- subset(Sg.all, phase=="greg")
Sg.greg$stadium <- factor(Sg.greg$stadium, levels = c("nymph", "adult"))
table.23 <- wilcox.test(AS ~ stadium, Sg.greg)
```

Table S23: Wilcoxon two-sample rank sum test results: *average speed* in gregarious final-instar nymphs and adults. Data from Martín-Blázquez and Bakkali (2017).

Test statistic	P value	Alternative hypothesis
1025	9.865e-05 * * *	two.sided

```
# temporary dataframe for plotting AS and AS_f in one figure
temp <- with(Sg.greg, data.frame(
  speed = c(AS, AS_f),
  kind = c(rep("raw", length(AS)), rep("norm", length(AS_f))),
  stadium=rep(as.character(stadium), 2))
  )
temp$stadium <- factor(temp$stadium, levels = c("nymph", "adult"))
temp$kind <- factor(temp$kind, levels = c("raw", "norm"))

# the infinite speeds in adults have yet to be removed
temp$speed[temp$speed==Inf] <- NA
```

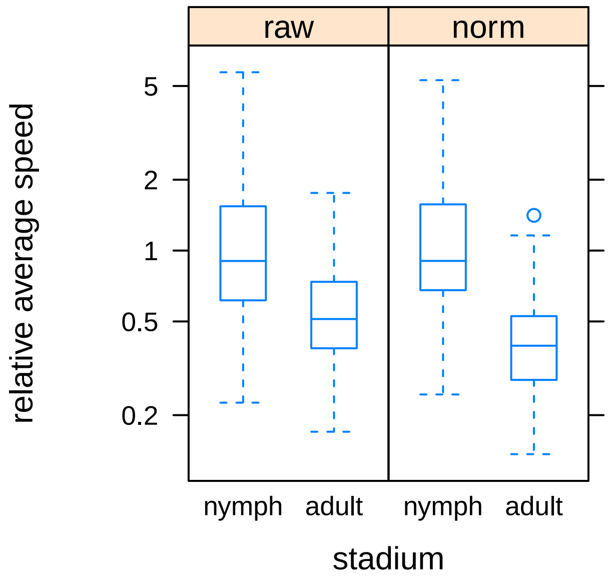

Figure S6: Difference in *average speed* between final instar nymphs and adults before ('raw') and after ('norm') division by *hind femur length*. The 'raw' and 'normalised' values are each expressed as fold-differences relative to the mean in nymphs. Data from Martín-Blázquez and Bakkali (2017).

```
# calculate speeds relative to mean in nymphs, separately in raw and norm
temp$rel.speed <- with(temp,
     ifelse(kind=="raw",
     speed/mean(speed[kind=="raw"], na.rm = TRUE),
     speed/mean(speed[kind=="norm"], na.rm = TRUE))
     )

# Figure S6
bwplot(rel.speed ~ stadium | kind, temp, pch="|",
       scales = list(y = list(log=T, equispaced.log=F)),
       ylab="relative average speed", xlab="stadium", layout=c(2,1))
```

Code for Figure 1

```
library(cowplot)
library(latex2exp)

set.seed(1122)
N=800
x1 <- runif(N, -.5, .5)
x2 <- runif(N, -.5, .5)

# calculate latent variable y' from x1 and x2
y.prime <- 2.5*x1 + 5*x2 + rlogis(N)

# binary outcomes A and B from y'
y.bool <- y.prime > 0
y <- c("A", "B")[as.numeric(y.bool)+1]

XY <- data.frame(x1, x2, y)

# Panel (A): Density histogram for x1
unif.x1.d <- ggplot(XY) +
  geom_histogram(aes(x=x1, y=..density.., color=y, fill=y), size=1, binwidth = 0.1, boundary = 0.5, pos
  scale_color_manual(values=c("deepskyblue2", "black")) +
  scale_fill_manual(values=c(alpha("deepskyblue2",0.4), alpha("black",0))) +
  scale_x_continuous(breaks=c(-.5, 0, .5)) +
  scale_y_continuous(limits = c(0, 2), expand = c(0,0)) +
  xlab(TeX("$\\mathit{t}_1$")) +
  labs(fill=TeX("$\\mathit{E}"), color=TeX("$\\mathit{E}"))

# Panel (B): Density histogram for x2
unif.x2.d <- ggplot(XY) +
  geom_histogram(aes(x=x2, y=..density.., color=y, fill=y), size=1, binwidth = 0.1, boundary = 0.5, pos
  scale_color_manual(values=c("deepskyblue2", "black")) +
  scale_fill_manual(values=c(alpha("deepskyblue2",0.4), alpha("black",0))) +
  scale_x_continuous(breaks=c(-.5, 0, .5)) +
  scale_y_continuous(limits = c(0, 2), expand = c(0,0)) +
  xlab(TeX("$\\mathit{t}_2$")) +
  labs(fill=TeX("$\\mathit{E}"), color=TeX("$\\mathit{E}"))

# Fit LR model to the simulated data
( m <- lrm(y ~ x1+x2, XY, x=T, y=T) )

XY$p <- predict(m, type = "fitted")
latent.slope <- m$coefficients["x2"]/m$coefficients["x1"]

# Panel (C): Scatterplot with latent axis
unif.xy.scatter <- ggplot(XY, aes(x1, x2)) +
  geom_point(aes(colour=p, shape=y), size=1.5, stroke=1, alpha=.7) +
  scale_shape_manual(values = c(4, 21)) +
  scale_colour_gradient2(low="deepskyblue2",
                         mid = "black",
                         high="orange",
                         midpoint = .5) +
```

```
  coord_fixed() +
  guides(shape = guide_legend(order = 1)) +
  geom_abline(slope=5/2.5, intercept=0, colour="black", linetype="dashed") +
  geom_abline(slope=latent.slope, intercept=0, colour="black", size=0.75) +
  scale_x_continuous(breaks=c(-.5,0,.5)) +
  scale_y_continuous(breaks=c(-.5,0,.5)) +
  xlab(TeX("$\\mathit{t}_1$")) + ylab(TeX("$\\mathit{t}_2$")) +
  labs(shape=TeX("$\\mathit{E}"), color=TeX("$\\mathit{P}(\\mathit{E}=B|\\mathit{T})"))

# Panel (D): Distribution pf P(E|T) in A and B
unif.p.box <- ggplot(XY, aes(x=y, y=p)) +
  geom_violin() +
  geom_boxplot(alpha=0.5) +
  ylab(TeX("$\\mathit{P}(\\mathit{E}=B|\\mathit{T})")) +
  xlab(TeX("$\\mathit{E}"))


fig.1 <- ggdraw() +
  draw_plot(unif.xy.scatter, 0, -0.05, 0.625, 0.625) +
  draw_plot(unif.p.box, 0.63, 0, 0.35, 0.5) +
  draw_plot(unif.x1.d, 0, 0.55, 0.5, 0.45) +
  draw_plot(unif.x2.d, 0.5, 0.55, 0.5, 0.45) +
  draw_plot_label(c("A", "B", "C", "D"), c(0, 0.5, 0, 0.63), c(1, 1, 0.5, 0.5))

save_plot('Fig1.pdf', fig.1, base_width = 7.086614, base_height = 6)
save_plot('Fig1.png', fig.1, base_width = 7.086614, base_height = 6)
```

References

Feltz, C J, and G E Miller. 1996. “An Asymptotic Test for the Equality of Coefficients of Variation from K Populations.” *Statistics in Medicine* 15 (6): 647–58. doi:10.1002/(SICI)1097-0258(19960330)15:6<647::AID-SIM184>3.0.CO;2-P.

Martín-Blázquez, R, and M Bakkali. 2017. “Standardization of Multivariate Regression Models for Estimation of the Gregariousness Level of the Main Pest Locust.” *Entomologia Experimentalis et Applicata* 163 (1): 9–25. doi:10.1111/eea.12564.
